# The Doctrine of Poisons

**Published:** 1873-12-15

**Authors:** Hiram Nance

**Affiliations:** Kewanee, Ill.


### THE DOCTRINE OF POISONS.

AN ESSAY READ BEFORE THE MILITARY TRACT MEDICAL ASSOCIATION, AT GALVA, JULY 8, '73.

BY HIRAM NANCE, M.D., OF KEWANEE, ILL.

Medical science is so full of matter that is both scientific and practical, that a physician who has been in practice even for a few years should be able to give a synopsis of his every-day experience that may be of some interest to his professional friends. And as our Association only meets semi-annually, it behooves us to make our essays and discussions as practical as we conveniently can.

I wish to call your attention to the subject of Toxicology, or the Doctrine of Poisons, and more especially cases of poisoning that have come under my own care during my professional life. I am quite assured that this subject does not meet with the attention that it deserves; and that many cases of poisoning, in every community, are treated as ordinary diseases and as originating from unavoidable causes, when, if the facts were known, poison in some way would be the cause. Poisons, in an indirect way, gain admission into the system very frequently—such as the poison of small-pox, scarlatina, typhus and typhoid; and all of those diseases are supposed to be of a contagious character. But of this character of diseases it is not my purpose to treat, but shall direct your attention to specific poisons as defined by Taylor. He says a poison is a substance which, when admitted in small quantity, is capable of acting deleteriously on the body. This definition, though usually and commonly explained, has its objections, as poisons may be used in either large or small quantities and produce their toxic effect.

We should all be on the alert when called to a person taken suddenly ill after having eaten anything, for upon our diagnosis frequently depends the life of our patient. I think, in cases of illness occurring suddenly, where the patient is vomiting, purging, with tenesmus, pain, etc., and during an epidemic of cholera morbus, diarrhœa, and other gastro-intestinal diseases, that we are too apt to be satisfied that the disease is idiopathic, and not depending upon the maladministration of any deleterious agent. As a physician, pathologist, or medical jurist, we are all interested in the peculiar effects of poisons. The physician must know the properties of his medicines, especially those remedies usually styled *heroic;* and without *heroic* remedies, what is our profession but a mere bagatelle—a mere game of chance; one that you can *cut* and *dry?*

But, gentlemen, we claim that though many imperfections may surround our beloved profession, that we are not *dead-heads*, but that a *halo* of science encircles the calling which we have made our choice. If we repudiate all our valuable and thoroughly-acting agents, we run into an expectant plan of treatment, and our science lapses into an infinitesimal, or homœopathic humbug. I mean by this that our valuable remedies, in chemistry and materia medica, are really not poisons, but that most of them, by maladministration, can be made such. It seems to be a law of nature that all good things, if improperly administered or used, are followed by a penalty; and this law equally applies to medical agents. How necessary, then, is it for us, as practicing physicians, to carefully study the effects of medicine on the system in large and small doses, and to carefully draw the distinction and diagnostic marks between ordinary disease and the effects of poisons. I am aware that many diseases so closely resemble the effects of poison that it is extremely difficult for even an experienced adept in toxicology to correctly diagnose the difference; but let us study this science carefully and thoroughly and we will save many lives, and not disgrace ourselves as medical jurists before the scrutinizing barrister, or learned court. You have all felt chagrined and humiliated, in sympathy with our medical or surgical experts put on the witness-stand; and in no place can a physician gain more applause, or receive the denunciations and opprobriums of the profession and public, than in appearing before a judicial tribunal in a case where one of our peers is arraigned for poisoning one of our fellow-men. Then, gentlemen, you see the importance of thoroughly understanding the subject of toxicology, and being ready at all times when called to a patient, if there is anything peculiar in his or her symptoms, to at least clandestinely suspicion the administration of poisons. In order to be prepared to meet those cases as they arise, we must not be as the lawyer, who, when consulted by his client in a case of assault and battery or malpractice, can tell him: "Well, call to-morrow, and I will have your case looked up." No, this won't do; we must be minute-men! ready at all times to make the diagnosis and found the treatment. An inability thus to act probably results in the death of our patient, and to our public disgrace. If not publicly disgraced, and the community are disposed to throw around us the mantle of charity for our ignorance, yet this want of knowledge keeps coming up in our minds, and the thought is constantly presenting itself: Well, I ought to have known more. I ought to have been better posted.

Cases of poisoning occur in all communities much more frequently, I think, than is generally supposed. It is no unusual thing to hear of a sudden death from vomiting and purging, from apoplexy, from chronic diarrhœa, from dyspepsia and diseases of the stomach, from sudden inflammation of the bowels, from hysteritis, and peritonitis. Sometimes our subject is found cold and rigid in the bed in the morning; sometimes the features turgid and apoplectic; sometimes the countenance remains placid, and the features but little changed. Now, gentlemen, any of those persons described may have died from the effects of ordinary disease, produced by ordinary causes; or they may have died from the secret administration of some of our medicinal agents.

Arsenic, in the form of *arsenious acid*, from its almost entire insipidity, has been most usually selected by the murderer to do his work. He can administer it in small doses, rarely producing vomiting, but acting slowly and insidiously on the mucus tissue of the stomach, the person to whom the poison is being administered rarely even suspecting anything being administered to his detriment. And when he calls to see his learned physician, he tells him: "Well, sir, you have dyspepsia, indigestion, or a kind of chronic gastritis; and by care in diet, and a little light medicine, you will soon be well." Thus the case goes on from day to day, from week to week, until the poor patient wears out with his dyspepsia, and death closes the scene, and no one, not even the doctor, suspected anything wrong. Those cases occurring in Washington City during the short administration of President Taylor, called the "White House Poisoning," were something of this kind. Though no proof, I believe, was ever produced to prove that a process of slow poisoning was practiced, yet most persons felt quite convinced that this was the case. There are two views held by toxicologists in regard to arsenical poisoning by the slow process: one holding that the poison, in small doses, is *not* cumulative, and the lesser number advocating the view that its long-continued use, in small doses, *is* cumulative, and that all of a sudden the specific action of the poison may show itself by vomiting, purging, great prostration, coldness, lividity of the surface, sunken eyes, frequent small pulse, a peculiar husky voice, leading rapidly to death.

A few years ago, while practicing in Stark county, a gentleman called on me just at evening, urging me to call and see his family—five of them, wife and four children. I was somewhat startled on his informing me that so many were sick, and inquired what they had ate for dinner. He said nothing unusual. I then asked him if he ate dinner at home; he said not—he was perfectly well. I now started to the house to see them, he accompanying me. On the way, I inquired if any other persons had dined with them; he said yes. Now, said I to him, we will hurry to your house, and then you send a messenger to the house of the lady that dined with your family, and see if she is not suffering from the same symptoms that your family are. The messenger was sent, and soon returned, stating that she was *very ill*—vomiting, purging, and all the ordinary symptoms of arsenical poisoning which I have described.

I now felt convinced that the family were poisoned, as all who partook of the dinner were very ill. I diagnosed arsenical poisoning; but to prove my diagnosis, and apply my antidote, was the next thing. I could easily give what I supposed to be the antidote, and was so well convinced that I was correct, that I immediately administered the hydrated sesqui oxide of iron to them all, and sent some to the young lady who was so unfortunate as to eat of the good dinner. Am happy to state all recovered. But my mission was not done until I was fully satisfied where this poison came from. I made scrutinizing inquiries of Mrs. White, and learned that no one but the family and the young lady had been about the house that day. No provisions of any kind had been sent in by any person. No unusual diet of any kind had been indulged in, with the exception of mince-pie. Here, I thought, I had my case. But how could mince-pie cause arsenical poisoning? The pie was produced; nothing unusual about its appearance or taste; but the plate, a common queensware or potters' plate, was cracked in a thousand little places in the glazing. I made rigid inquiries as to what she could have had on the plate; but it was not until the next day that the thought presented itself to her that the autumn before she had been using *fly-poison* on the plate—*cobalt*—which contains, usually, a large amount of arsenious acid. The poison had settled down in the little cracks, and in baking the pie the deleterious agent had diffused itself in the pie. The question now arises: What if my diagnosis had been incorrect, and I had not given the antidote, what would have been the result? Any of your judgments would be equally as good as mine in this case. Would enough arsenic be absorbed in the fissures of the plate, and be expelled by the heat into the pie, to cause death in any of those cases, had the antidote not been given? They might all have recovered; but I would prefer eating my pie, and feeding my children on pie, not baked on arsenical plates.

A few years after seeing those cases of arsenical poisoning, I was called in the country, four or five miles distant, to visit a family suffering in the same way. Symptoms not unlike Asiatic cholera; great prostration; eyes sunken and turgid; profuse sweats; almost constant vomiting and diarrhœa; hoarseness; small, quick pulse, etc. There were five or six in the family, all sick but the father. As cholera was not prevailing, I could not compare the disease to anything but arsenical poisoning. Gave the usual antidote, and my patients all finally recovered, but not for some days. The same family, months afterwards, suffered in the same way. I was called again, and gave the same treatment. And I learned, after I left that community, that they had another attack, and a physician from this place was called. What his diagnosis and treatment were I never learned.

But the peculiarity in the case was, that the husband and father never suffered any of those attacks; and I believed him to be the guilty party in the administration of the supposed poison; and on making inquiries it seems that he had formerly been insane in the Eastern States, before emigrating here; and I believe he possessed an insane impulse, or monomania, on poisoning his family.

The carelessness of leaving arsenic and other poisons lying around in convenient places for persons to get hold of, and without any mark or label, should be sufficient cause for legal action; and the person thus found guilty should be punished to the amount of damage sustained; and in case the poisoning results in death, then the poisoner should be punished as any criminal guilty of murder in the second or first degree. We, as physicians or druggists, are held responsible for the maladministration of medicines, or for an error in writing a prescription, or error in filling the same; and why should any sensible man or woman not be held to the same accountability?

In this connection I refer you to the case of the Rev. Mr. Wolliscroft, a former highly educated minister of the M. E. Church, who preached in this locality some fifteen or twenty years ago. He had been traveling all day. Called on friends in Peoria, and enjoyed a good supper with them. On going to bed he made the remark to the good lady of the house that he had a *sour stomach.* She remarked she had some magnesia which would soon remedy that, and gave him what she called magnesia. He partook of it plentifully and went to bed. In three or four hours he was waked up vomiting. He called his friends to him fearing something was wrong. The lady became alarmed, looked to her magnesia and found it was arsenious acid. Next day the good man was a corpse. I look upon this as *legal* carelessness, and not to be excused only before the prosecuting attorney, judge and jury. If one of the citizens of Galva is prosecuted for permitting a physician to come in his drug-store and taking or giving to one of his associates extract of belladonna instead of extract of taraxacum, and the good jury see fit to fine him for damages sustained because he is a druggist, then why not punish with equal severity any person or persons permitting poisons to lie around without any mark or label? I am happy to state that, either by general consent or legal enactment, our druggists are now all fixing the cross-bones on every poison that leaves their stores; and the law of our State is such that no package can leave a druggist's store without the physician's inscription on it, in case of prescription, or, when ordered by the bearer, the name of the medicine. This is certainly a step in the right direction; and now let us make the responsibility rest on the non-professional as well as on us.

Poisoning from opium and its various preparations is so common that I presume few of my medical friends have not seen cases of it. I call your attention to a case I attended some years since: A Swede, residing six or eight miles away, called on me in the latter part of the night, saying his brother (a young man) was suffering from severe pain in the bowels. I prescribed and sent him three or four powders, each containing about ¼ gr. morphine, ordered them given every two or three hours, until relieved, and if not better by 7 or 8 o'clock in the morning, to send for me. He returned before the time appointed, but instead of saying his brother was in pain, said he was so stupid he could not be aroused under any circumstances. I suspicioned *opium poisoning;* and as he was one of those *half-fool* doctors himself, I suspected he had been giving opium before he came for me. My suspicion was correct, as he acknowledged giving him two or three teaspoonfuls of a preparation of opium and whisky, which he had formerly got and prepared himself and kept in the house. Found the patient in a complete comatose state. Pulse about 100

or 110; respirations about eight or ten; profuse sweat; countenance purple and turgid; heart beating as if he had hypertrophy of this organ; eyes about half closed, and no treatment could induce him to open them. I immediately bled him copiously. Gave him sul. zinci as an emetic, but it produced but little effect. Removed all clothing but shirt and drawers; applied sinapisms to feet and legs; poured down hot coffee, and raised him in an erect position, and kept him in motion, by a man on each side of him occasionally using flagellation by using keen switches to the surface. I sent for a physician to assist me. The treatment was not changed, but continued by us for thirteen hours before the effects wore off sufficiently for him to speak. He recovered perfectly.

The wife of a very near friend and relative of mine found her fine little babe in the night a little pained and restless. She got up and gave it half a teaspoonful of what she thought was *paregoric*. The babe slept sweetly until morning, then awoke her by its heavy breathing, and immediately went into convulsions, and by breakfast time it was a corpse. The medicine given proved on inspection to be tincture of opium, hanging up by the side of the paregoric bottle.

J. M., living six miles from Kewanee, called on me to see his baby, aged about one year. When I got there found it dying from the effects of opium. The father and mother had been giving it a quack preparation of Dr. Janes', in doses six or eight times the amount specified in the instructions.

Of the *treatment*, but little more can be added. Emetics of sul. zinci or sul. copper, stomach-pump, cold douches, coffee, decoction of galls, and recently belladonna in full doses, and by hypodermic injections, have been used. I have no experience with the last-named remedy.

I have seen one or two cases, in my early practice, of partial poisoning from my own administration of tartarized antimony, in cases of *croup*, my great anxiety to relieve my patient leading me to the too frequent and bold administration of this *heroic* and powerful remedy. The symptoms are: prostration; constant vomiting, or rather continued nausea, followed by vomiting the contents of the stomach; then glairy fluid and bile; copious sweats; eyes sunken; skin partially corrugated, etc. In fact, the symptoms resemble those in epidemic cholera. The treatment: infusion of cinchonia in large quantities; tannic acid; infusion of galls, or powdered cinchonia bark, or anything containing the properties of tannin, as oak-bark infusion.

Fifteen or twenty years ago I used to see cases of poisoning from rattlesnake bites. A Mr. B., living five or six miles from Galva, was bitten, on a very warm day in July, on the finger. He had picked up a sheaf of wheat containing the serpent. I was sent for, but not being at home my place was supplied by an *eclectic*. He was discharged after thirteen hours, and I called. Found him suffering severely; swelling extending to the breast neck, etc.; great prostration and anxiety; occasional vomiting; the finger and hand took on *sphacelus;* and in a day or two he was dead. Had he not been bitten on the finger (so sensitive an organ), and during the heat of the day, when his blood was more than up to fever-heat, he might not have died. No other case that came under my care died. I gave them whisky or brandy in large quantities, and ammonical liniments applied to the swollen parts. An antidote within ten or twelve years has been proposed and used, called Bibron's Antidote, composed of

Iodine, grs. iv.,
Corros. chloride of mercury, grs. ii.
Bromine, f. ℥ v.—M.

Dose: ten drops in a little wine or brandy, to be repeated as often as necessary. I have never since its introduction had occasion to use it.

I would not be performing my duty faithfully to the Society, in this essay, were I to omit calling your attention to the frequency of poisoning from the caustic preparation used in almost every house by our lady-friends in the making of soap. Concentrated lye, soap-makers' potash, lye from ordinary lixiviation from wood-ashes, etc., all have their victims; and I have been called to see cases of poisoning from all of them. Usually find the poor little child with lips and mouth swelled, excoriated; inability almost to swallow; abrasion of the mucus membrane of the lips and mouth. If death does not result soon, which rarely happens, the little patient becomes distressed terribly by pain and ulceration of the stomach and bowels. Diarrhœa, etc., continues for weeks, months, and sometimes a year, before death closes the horrid suffering. Of course, if we saw the patient sufficiently early we would give the acids to counteract the effects of the alkalies, such as lemon-juice, vinegar, citric acid, or the fixed oils; would prefer the former, diluted with large quantities of water. But the mischief is usually done. In addition to this would give demulcent and mucilaginous drinks, and counteract the effects of the gastritis and ulceration of the bowels on general principles, including opiates, mild astringents, soothing applications to the bowels, etc.

Have seen several cases of poisoning from children eating the sweet and pleasant-tasting seed of the stramonium plant. The symptoms are strange and interesting in the extreme. The little patient is in constant motion, picking at imaginary objects constantly, and continually running over some language unlike English, Dutch, Latin, Modoc, or anything else familiar to a good linguist. Recovery usually takes place. I give emetics of sul. copper, repeated until I cannot see any more seed ejected. Opiates to allay restlessness, and infusion of the astringents in full doses are recommended.

Was called to see a girl aged about six or eight years. Found her suffering from severe pain in the stomach and bowels; constant tenesmus, and dysenteric discharges containing a large amount of mucus and blood. There was no dysentery prevailing at the time—and at the time of year that we rarely see it—and the case presented more prostration and cholera-like symptoms than are usually seen in either sporadic or epidemic dysentery. I could find no clew to any poison being used; but after the death of the little girl, another child remembered being in the cellar, and saw the dead take from a shelf some white lumps looking like sugar. On examination it was found to be sugar of lead. Antidotes for lead: sul. soda or sul. magnesia, mucilaginous drinks, albumen, milk, and chloroform to ease the pain.

Was called to see an English family, residing in an adjoining town, in the summer of 1868. Found the mother, grandmother, and five children of different ages, all very sick. Vomiting, tenesmus and purging; great prostration; sunken features; sweats; pain in the stomach and bowels; in fact, the symptoms resembled epidemic cholera: and if this disease had been prevailing at the time I might have diagnosed them as such. But I suspected poison, and made the inquiry. The family were a very ignorant one, and carelessness and indifference seemed to be their prevailing traits, and it was some time before I could get them to acknowledge eating anything unusual. Finally the mother said the boys had been picking *mushrooms*, and they had all ate of them who were sick. There is no direct antidote. Emetics should be given, if the substance is not already expelled, and chloroform to ease the pain. I gave opiates, applied epispastics of mustard, fomentations to the bowels, etc. All recovered.

In cases of poisoning from *strychnia*, brucia, and all the preparations of *nux vomica*, the physician rarely arrives in time to be of any service to the patient; usually finding the person dead. The symptoms are so well-marked during the stage of the toxic effect of the medicine or poison, that it does not require an expert to diagnose the disease. Spasms of a tetanic form usually make their appearance in from fifteen minutes to half an hour after the administration—rigidity of the extremities; subsultus tendinum; great pain in the stomach; difficult respiration; convulsions, and death. When death takes place the person is found almost immediately rigid, and so stiff that it is with difficulty a limb can be extended or flexed.

Was called to see a lady a few years ago (who had died very suddenly), and requested to make a post-mortem examination. Found her in the condition above described. She was apparently well, and in half an hour was dead. Circumstances were such that strong suspicion rested upon a relative of hers as being the guilty party. Post-mortem showed rigidity; gestation advanced to five or six months. I was convinced that she had taken strychnia. The prisoner was arrested. Strychnia was found in the house, and I analyzed, or rather tested it, before the circuit court of this county. The jury were convinced that she died from its effects; but the proof against the criminal was not sufficient to convict, the doubt with the jury being whether she took it herself, or whether he gave it to her. Formerly it was very difficult to detect strychnia or any of the vegetable poisons in the stomach on analysis after death; but recently our chemists are succeeding better, the tests showing plainly the poison.

Was called, about three years ago, to see James Fell, living near Cambridge. He had been under two physicians' care for two or three days before I saw him. Found him sitting up in bed in an emprosthotonos form, and as stiff as a board; impossibility to lie down. Tetanic convulsions every fifteen, twenty or thirty minutes; profuse, exhausting sweats, and almost complete insomnia; urine very scanty and high-colored; mind good. I ordered free and full doses of morphine with nitrate of potash, chloroform, etc., to ease the pain and procure sleep; mucilaginous drinks, m. tinct. iron and quinia as a tonic. Case continued a week or ten days; convalescence came on, and he slowly recovered. The poison got admission into his system in one or all of three ways: He had been planting corn, and had mixed the corn in a solution of strychnia to kill squirrels, gophers, or birds. He put the corn in his pocket, and probably in the same pocket put his quid of tobacco, and in chewing received the poison. Or, the corn being wet, the poison might have been absorbed by hypodermic method; or, lastly, he had some sores on his foot, and he might have put his hands on the abrasions, and it been received in this way.

Probably the best treatment in cases of poisoning from these preparations of nux vomica, is *chloroform* in drachm doses, tannic acid, or other astringents, mucilaginous fluids, etc. There is no direct antidote.

Two years ago I was sent for to see Mr. R. R., living about one mile from town. Found him vomiting profusely of blood and mucus; pulse very weak; respiration laborious; muscles of face convulsed; limbs cold and damp; sense of burning in the stomach. He had taken, half an hour before, one spoonful of nit. potash, supposing it to be sul. of magnesia. The peculiarity of the case was the large amount of blood and mucus ejected from the stomach. Taylor says this only occasionally occurs. There is no direct antidote. I gave opiates to ease pain, and mucilaginous drinks. The patient recovered.

Last summer Mrs. G. called on me for treatment for leucorrhœa. I gave her the ordinary internal treatment, and put her up a paper of medicine containing sul.zinci, to be used for injection per vagina. A week or two afterward I was called to see her suffering from continued nausea and profuse vomiting; great pain in the stomach and bowels; profuse sweats, and pale countenance. As it was at a time when we were having bowel and stomach diseases, I suspected nothing unusual, and prescribed a cholera mixture containing chloroform, tinct. opii., tinct. camphor, tinct. capsicum, and alcohol, to be given in teaspoonful doses every fifteen or twenty minutes. This eased the pain and checked the vomiting; but she remained sick, and the stomach was very irritable, for several days. She recovered, and afterward confessed to me she had taken the white vitriol in a teaspoonful dose, supposing at the time it was salts.

Mrs. Moody consulted me, a few years ago, in regard to a disease requiring the external application of oil tiglii. I gave her a prescription for two bottles of medicine, one containing croton oil, the other some medicines to be taken internally. The druggist did his duty, carefully writing on each bottle *how to be used;* but the patient, notwithstanding this, took the wrong bottle containing the croton oil. She had taken one teaspoonful containing equal parts of croton and olive oils. I found her continually purging, and in great pain in stomach and bowels; vomiting, prostration, sweats, etc. Gave her opiates, mild drinks, and fomentations to bowels and stomach. She recovered.

Four or five years ago I was solicited to make a post-mortem examination in the case of E. H., aged about 34 years. He had been suffering from ophthalmia and granulated lids. Had formerly been under my care, but not getting along as fast as was desirable, he became acquainted with a notorious quack, or empyric, who learned of his sore eyes and called to see him, and by his flattery and fair promises induced him to accompany him to Toulon, procure a boarding-place, and put himself under his medical care. He was there but a few days, and then commenced vomiting occasionally—diarrhœa, and great prostration—so much so that a neighbor of his speaking to me about him, said that he looked so strangely that his own friends hardly recognized him. A few days after this the post-mortem was made by Dr. Scott, of Galesburg, and Dr. Smiley and myself. We found the stomach, duodenum and ileum, all very much inflamed, and of a deep and beautiful scarlet color. We found nothing in the stomach or bowels except a very mucous fluid with a few little pearl-like shining substances resembling oyster-shell. We all felt pretty well convinced that he had died from the effects of some corrosive poison; and what lead to the suspicion still more was, that the quack had made some trade with him, and if he died no one would know the amount that he had received. I took the stomach and part of the bowels, sealed them up, and expressed them to Dr. Mahla, of Chicago, with a request that he analyze them. He wrote me that he would not do it for less than one hundred dollars. I spoke to the friends about it, and they said they had not the money. I then wrote the doctor that I would see it paid, and referred him to some national banks as reference. He wrote me again that he would not touch it without the cash in advance. This the friends refused to raise, and consequently no analysis was made, and the matter was dropped, and the quack not prosecuted.

In conclusion, I would say that every physician residing in country places where *antidotes* cannot be readily obtained at short notice, should keep on hand antidotes for the usual poisons, plainly labeled, and also for what poisons they should be administered. Stomach-pumps, if used to remove the poison, should be carefully introduced, and manipulated with care, or we may seriously injure the mucous tissue of the œsophagus or stomach. As a rule, when called to a patient taken suddenly ill after something has been eaten, we should suspect poison, and act accordingly; and if we feel sufficiently convinced, then apply our remedies. In nearly all cases we should give emetics, mucilaginous drinks, albuminous substances, milk, etc., and then, as soon as we are fully satisfied, administer our specific antidote.

As any of those cases may result in a *medico-legal* investigation, it is our duty, as medical experts or jurists, to reserve all ejected matter, or anything else that may lead to the detection of the poison and conviction of the criminal. Poisons found about the house, or parts of the viscera retained for analysis, should be carefully kept by one person, and sealed and locked up until in the hands of the chemist.

